# The morphology of maxillary first and second molars analyzed by cone-beam computed tomography in a polish population

**DOI:** 10.1186/s12880-017-0243-3

**Published:** 2017-12-29

**Authors:** Katarzyna Olczak, Halina Pawlicka

**Affiliations:** Department of Endodontics, Medical University of Lodz, Poland, Pomorska 251, 92-213 Lodz, Poland

**Keywords:** Canal morphology, Cone-beam computed tomography, Maxillary molar, Root morphology

## Abstract

**Background:**

The success of endodontic treatment is greatly affected by the location of the root canals. The purpose of this study was to evaluate the root and canal morphology of permanent maxillary first and second molars in a Polish population using cone-beam computed tomography scanning.

**Methods:**

Cone-beam computed tomography (CBCT) scans of maxillary first and second molars the maxilla were examined. The number of roots and root canals, and the frequency of additional canals (MB2) in the mesiobuccal root canals were determined. The results were subjected to statistical analysis using the chi-square test or the chi-square test with Yates’ correction.

**Results:**

A total of 112 CBCT images of maxillary first (*n* = 185) and second molars (*n* = 207) from 112 patients were analyzed. All the maxillary first molars had three roots (100%). The majority of maxillary second molars had three roots (91.8%), 5.8% had two roots and 2.4% had one root. A statistically significant difference was observed between the numbers of roots in the maxillary first and second molars (*p* < 0.01). A statistically significant difference was also found in the distribution of the number of canals in the maxillary first and second molars (*p* < 0.001). The majority of maxillary first molars had four root canals (59.5%), while 40.5% had three root canals. Most maxillary second molars had three root canals (70%). Additional canals (MB2) in the mesiobuccal roots were detected significantly more frequently in the maxillary first molars than the second molars (*p* = 0.000) and more frequently in men than in women (*p* < 0.05). A higher prevalence of two canals in the mesiobuccal roots in maxillary second molars occurred in patients aged between 31 and 40 years than in patients aged between 21 and 30 years. In the maxillary first molars, the prevalence of the MB2 canal in the mesiobuccal root was almost equally distributed in the two age groups (21–30 and 31–40 years).

**Conclusion:**

Within the limitations of this study, it can be concluded that there are differences in the number and configuration of roots and root canals between maxillary first and second molars in the studied patients of a Polish population.

## Background

The success of endodontic treatment is greatly affected by the location of the root canals. The root canal system is surprisingly complex, as revealed by a number of studies of dental anatomy [1]. The internal morphology of teeth is a labyrinthine challenge for the dentist, who is required to make full use of any acquired knowledge and skills to avoid making mistakes during root canal treatment procedures. Root canals which are not identified during treatment become a reservoir for bacteria, thus preventing healing or allowing the formation of new inflammatory lesions in the periapical tissues [2]. The root canals of maxillary molars are particularly difficult to treat, being the most common examples of multiple roots and multi-root canals; however, other, less common forms of anatomical maxillary molars, such as teeth with only one root canal or teeth with C-shaped root canals, have also been described. Literature reports often emphasize the need to identify an additional root canal (MB2) in the mesiobuccal root; however, its incidence varies [3–5]. This variation can be attributed to the different methods that were used by the researchers: study protocols (in vivo or in vitro); sample size; and techniques used to identify canal configuration [3, 6, 7]. This variation could also be associated with age, sex, and ethnic differences of the study populations [8].

Since its introduction in 1990 to Endodontics, cone beam computed tomography (CBCT) has increased the potential for non-invasive analysis of internal and external dental morphology. In vitro and in vivo studies of computed tomography have significantly contributed to the understanding of craniofacial anatomy [7, 9].

### Aim

The purpose of this study was to evaluate the root and canal morphology of permanent teeth in a Polish population using cone-beam computed tomography scanning.

## Methods

All experimental procedures in this study were approved by the Ethics Committee of the Medical University of Lodz (Protocol n° RNN/166/15/KE). Cone-beam computed tomography (CBCT) scans of the maxilla of 112 Polish patients, taken as part of the diagnosis or planning of dental treatment in the period May 2015-December 2016 in the Dental Hospital of the Medical University of Lodz, were examined. The included CBCT scans presented first or second molars in patients between 21 and 40 year old.

The inclusion criteria were to have at least one 1st or 2nd upper molar with fully-developed apices. Teeth showing root resorption, root canal treatment, post or other crown reconstruction that would make difficult to assess their anatomy were excluded. Of the 300 CBCT scans examined, 112 fulfilled the above criteria.

All images were taken using a Gendex GXCB-500 ®machine (Gendex®) with image capture parameters set at 120 kV and 5.0 mA, and an exposure time of 11 s. The voxel size was 0.125 mm. The scans were analyzed using iCATVision software, version 1.9.3.13. All scans were evaluated separately by two endodontists and any disagreement was discussed until a consensus was reached.

The CBCT images were analyzed as follows. Axial, coronal, and sagittal two-dimensional sectional images were displayed. The number of roots and root canals, and the frequency of additional canals (MB2) in the mesiobuccal root canals were determined. It was also examined whether any relationships were present between the prevalence of the MB2 canal and the age and sex of patients. The results were subjected to statistical analysis using the chi-square test or the chi-square test with Yates’ correction.

## Results

A total of 112 CBCT images of maxillary first (*n* = 185) and second molars (*n* = 207) from 112 patients were analyzed. There were 74 women and 38 men with a mean age of 34,77 years (ranging from 21 years to 40 years).

All the maxillary first molars had three roots (100%). The majority of maxillary second molars had three roots (91.8%), 5.8% had two roots and 2.4% had one root. A statistically significant difference was observed between maxillary first and second molars regarding the number of roots (*p* < 0.01): Table 1.

**Table 1:** Number of roots in the maxillary first and second molars

Number of roots	Maxillary first molar		Maxillary second molar	
	n	Percent	n	Percent
1	–	–	5	2,4
2	–	–	12	5,8
3	185	100,0	190	91,8
Total	185	100,0	207	100,0

chi2 = 12,296; *p* = 0, 0021

**Fig. 1:**
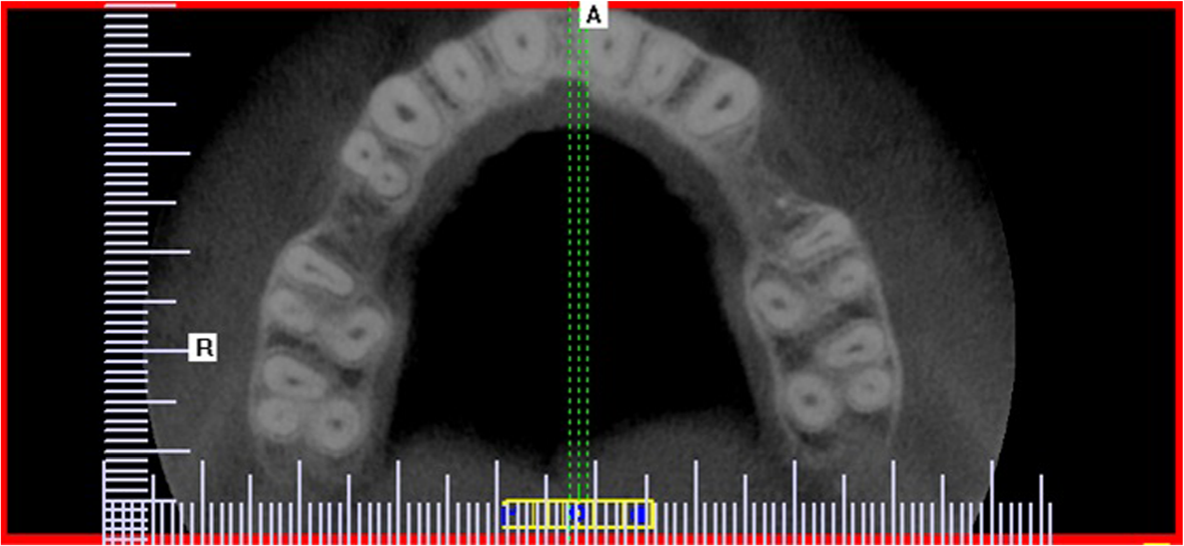
Cross-sectional CBCT image of maxillary first and second molars showing three root canals and three roots

**Fig. 2:**
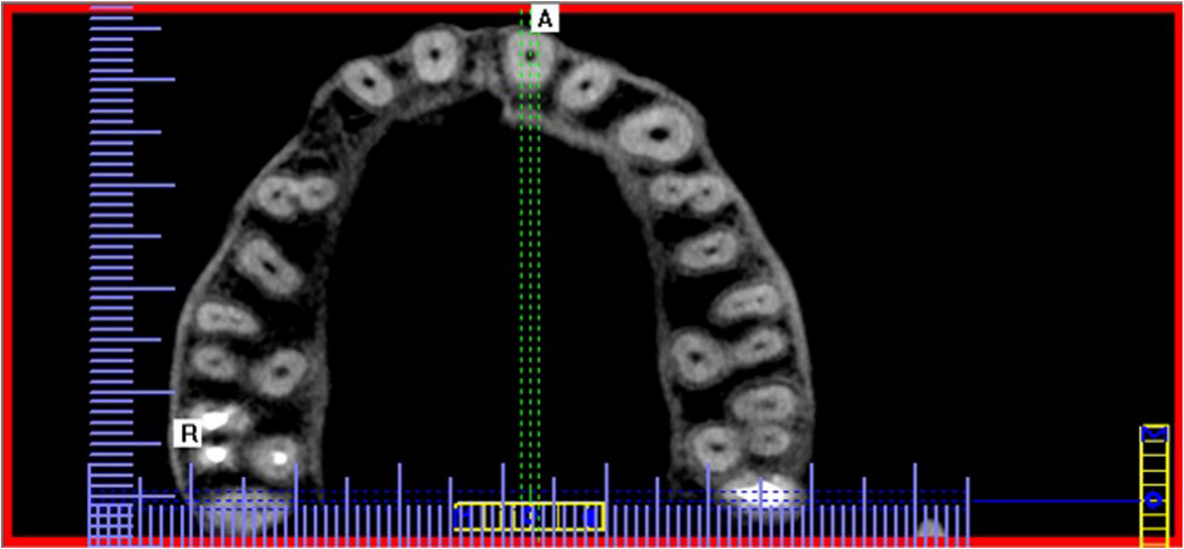
Cross-sectional CBCT image of maxillary first and second molars showing four root canals and three roots

**Fig. 3:**
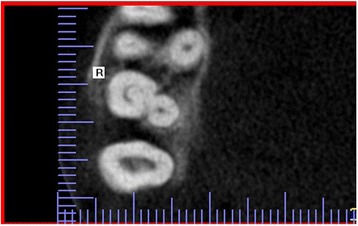
Cross-sectional CBCT image showing C-shaped canal configuration in maxillary second molar

A statistically significant difference was also found in the distribution of the number of canals in the maxillary first and second canals (*p* < 0.001). The majority of maxillary first molars had four root canals (59.5%), while 40.5% had three root canals. The presence of three root canals was significantly more common in the second than the first maxillary molars (*p* = 0.000). Most maxillary second molars had three root canals (70%). In other maxillary second molars, four canals (23.2%), two canals (3.9%), and one canal (1%) or C-shaped canals (1.9%) were observed (Figs. 1, 2, and 3 and Table 2). Additional canals (MB2) in the mesiobuccal roots were detected significantly more frequently in the maxillary first molars (59,5%) than the second molars (23,2%) (*p* = 0.000).

**Table 2:** Number of root canals in the maxillary first and second molars

Number of root canals	Maxillary first molar		Maxillary second molar	
	n	Percent	n	Percent
C-shaped canal	–	–	4	1,9
1	–	–	2	1,0
2	–	40,5	8	3,9
3	75	59,5	145	70,0
4	110	–	48	23,2
5	–	100,0	–	–
Total	185		207	100,0

chi^2^ = 52,601; *p* = 0, 0000

The MB2 canal occurred significantly more frequently (both in upper first and second molars) in men than in women: 68.6% vs 53.9%, respectively, in the maxillary first molars; and 34.7% vs 17.0% in the maxillary second molars (Table 3, Table 4). A statistically significant difference in the prevalence of the MB2 canal in the first and second molars was found between the groups of men and women (*p* < 0.05).

**Table 3:** Distribution of MB2 canals in maxillary first molar teeth according to patient’s sex

MB2 canal in maxillary first molar teeth	Sex				Total
	Female		Male		
	n	Percent	n	Percent	
MB2 present	62	53,9	48	68,6	110
MB2 absent	53	46,1	22	31,4	75
Total	115	100,0	70	100,0	185

chi^2^ = 3,878; *p* = 0,0489

**Table 4:** Distribution of MB2 canals in maxillary second molar teeth according to patient’s sex

MB2 canal in maxillary second molar teeth	Sex				Total
	Female		Male		
	n	Percent	n	Percent	
MB2 present	23	17,0	25	34,7	48
MB2 absent	112	83,0	47	65,3	159
Total	135	100,0	72	100,0	207

chi^2^ = 8,239; *p* = 0,0041

Regarding age, no statistically significant difference in the prevalence of the MB2 canal was found between the two tested age groups for the maxillary first molars; however, in the second molars, the MB2 canal was observed significantly more frequently in the group of patients aged 31–40 years than in those aged 21–30 years (Table 5, Table 6).

**Table 5:** The presence of MB2 canals in first maxillary molars according to patient’s age

MB2 canal in maxillary first molar teeth	Age (Y)				Total
	21–30		31–40		
	n	Percent	n	Percent	
MB2 present	28	58,3	82	59,9	110
MB2 absent	20	41,7	55	40,1	75
Total	48	100,0	137	100,0	185

chi^2^ = 0,34; *p* = 0,854

**Table 6:** The presence of MB2 canals in second maxillary molars according to patient’s age

MB2 canal in maxillary second molar teeth	Age (Y)				Total
	20–30		31–40		
	n	Percent	n	Percent	
MB2 present	9	18,0	39	68,4	48
MB2 absent	41	82,0	18	31,6	59
Total	50	100,0	57	100,0	107

chi^2^ = 27,382; *p* = 0,0000

## Discussion

The development of technology has made it possible for computed tomography to be used in the diagnosis and evaluation of endodontic dental anatomy. Various methods have been used for the analysis of internal dental anatomy, such as sectioning, canal staining and tooth clearing techniques, as well as radiographic techniques such as conventional and contrast medium-enhanced radiography. Although tooth-clearing techniques have been generally considered the gold standard for the evaluation of root canal morphology, these techniques are in vitro methods that use only extracted teeth; the clinical methods used for analyzing the internal anatomy of teeth are X-rays and tomograms [10].

CBCT offers significant advantages over X-rays [11]. While X-rays are limited by only being able to form two-dimensional images, computed tomography allows anatomical structures such as teeth and their neighboring structures to be observed in three planes. This allows for a very precise analysis of the construction of test items [12]. Of course in every situation, the good of the patient should be considered first and care must be taken for his or her safety. According to the principle of “primum no nocere” and “ALARA” (“As Low As Reasonably Achievable”) CBCT should be performed only when it is necessary and when it provides information significantly improving the process of diagnosis or treatment of the patient [13]. The CBCT scans used in the present study had been intended for diagnostic reasons, not only for performing scientific work.

The present study uses CBCT methods to make a thorough and comprehensive in vivo analysis of the root and canal morphology of the maxillary first and second molars in a Polish population. It was found that all of the maxillary first molars had three roots. These findings are consistent with those in Thai, Burmese and Kuwait populations [14–16]. In addition, other studies have shown that three-root form is found in over 90% of evaluated first molars [17, 18]. Zheng et al. [17] report that 97.29% of studied molars in a Chinese population had three separate roots, while a similar study in a Korean population found 97.91% of first maxillary molars to have three separate roots [19]. A higher incidence of variation in root morphology was found in maxillary second molars than first molars. Although up to 91.8% of the evaluated teeth have three roots, second maxillary molars were also found with two roots or just one root. Many studies have revealed more variation in the root number within the second molar than the first molar [17, 18]. Our results demonstrate a higher prevalence of three roots in second maxillary molars than reported in some earlier studies on Thai, Burmese and Indian populations [14, 15, 18]. These differences highlight the influence of ethnic background on tooth root morphology. The reason a high percentage of the second molars had three roots could be the lack of separate distinguishing fused roots.

In root canal treatment, the number and location of root canals is of greater concern to the dentist than the number of roots. The present study found that the majority of maxillary first molars had four root canals (59.5%), while 40.5% had three root canals. Other studies have found a high percentage of teeth to have four root canals [3, 19, 20]. In the present analysis, the first molars had three or four root canals. In contrast, studies on Chinese, Korean or Indian populations found a few cases of first molars with one, two, five, or six root canals, in addition to those with four or three root canals [21–23]. However these numbers of root canals were in the considerable minority and have usually represented no more than about 0.3–1.7% of inspected teeth [14, 15, 18, 21–23]. It is possible that these less common anatomical forms of molar teeth will also be identified in the Polish population in future studies based on greater numbers of teeth.

The second molar teeth presented greater diversity in the number of root canals, which has been confirmed elsewhere [19, 22, 23]. Amongst these teeth, it is possible to observe single-root canals and two-canals, as well as C-shaped root canals, which are very difficult to treat. In a study of a Chinese population using a clearing method, Yang et al. [23] report the presence of a C-shaped canal in 4.9% of maxillary second molars. In a CBCT study of a Korean population, C-shaped root canals were seen more frequently in the maxillary second (2.7%) than in the first (0.8%) molars [24]. In addition, a greater frequency of three-root canals, but a lower frequency of four root canals, was found in the second than the first molars. In all cases in the present study, the fourth root canal was found to be the mesiobuccal root second canal (MB2 canal). Very similar results were presented by Nikoloudaki et al. [25], who evaluated the morphology of upper molar teeth in a Greek population and found statistically significant differences in the distribution of root canals between maxillary first and second molars. In addition, the fourth root canal in the first upper molar was always the MB2 root canal, as in our present article. The MB2 canal was observed in 53.2% of maxillary first molars in the Greek population [25]. Tanavi et al. [20] report the percentage of MB2 to be 55.72% in maxillary first molars and 17.39% in maxillary second molars. Other studies have reported a higher frequency of additional mesiobuccal root canal [6, 7, 26–29]. Abarca et al. [6] found the frequency of the MB2 canal in a Chilean population to be 42.8% in the second molars and 73.44% in maxillary first molars. A similar high frequency (70.6%) of the MB2 canal was detected in another study based on scans of freshly extracted maxillary molars [7]. In an in vitro study of a Turkish population based on the clearing method, 93.5% of maxillary first molars were found to have two or more canal systems in the mesiobuccal root [27]. Laboratory studies by Kulid [30] and Gilles et al. [28] also note a high prevalence of the MB2 canal (96% and 90% respectively). A study on an Irish population found a higher occurrence of the MB2 canal both in first (78%), and in second (58%) maxillary molars compared to the present study [29]; however, a clinical study on a Saudi Arabian population found a low frequency of MB2 in second molars (19.7%) [31]. CBCT examinations revealed the presence of MB2 in the maxillary second molar in about 22% to 48% of teeth [4, 22, 32]. Generally speaking, laboratory-based studies identify greater numbers of roots and root canals than in vivo studies [33]. Despite this, a study by Pecora et al. [34] of 120 investigated teeth based on clearing identified the presence of a single root canal in the mesial root of maxillary first molars in 75% of the examined teeth. Two or more canal systems were observed in only 30 teeth.

The need to identify and treat the MB2 canal has a huge impact on the outcome of endodontic therapy [35, 36]. This root canal is often undetected and consequently becomes a cause of inflammatory lesions in the periapical tissues [36]. Shetty et al. [36] report the incidence of the MB2 canal as over 80% in maxillary first molars and almost 30% in maxillary second molars. The majority of maxillary first molars (77.19%) and maxillary second molars (90%) had an unfilled MB2 canal. Periapical radiolucencies were found in unfilled MB2 canals in 72.7% of maxillary first molars and 88.8% of maxillary second molars [36].

A number analyses of the frequency of the MB2 root canal depending on the age and sex of the patients based on CBCT scans have returned different results [6, 8, 20]. Our present study shows a significant relationship between sex and the incidence of the MB2 canal in maxillary first and second molars. However, it is worth noticing that the probability of error in the case of maxillary first molars was nearly equal 0.05 (*p* = 0.0489), thus the difference was close to the border of statistical significance. In maxillary second molars, the *p*-value was equal to zero (*p* = 0,000). Jin-Hee Lee et al. [32] report the prevalence of the MB2 canal in the mesiobuccal root of maxillary first molars to be almost equally distributed in groups of males and females, but also found statistically significant differences for the occurrence of second molars: 48.7% of MB2 in males and 30.8% in females [32]. Betancourt et al. [5] found that the MB2 canal in maxillary second molars was significantly more frequent in men that in women (*p* = 0.001). Sert and Bayirli concluded that sex was an important factor affecting the occurrence of the MB2 canal in a Turkish population: a single canal in the mesiobuccal root occurred only in 3% of males compared to 10% in females [27]. In contrast, the incidence of additional MB2 in other studies did not differ with regard to the sex of the patient [6, 8, 17].

The outcomes of studies on the correlation between the occurrence of the MB2 canal and patient age also vary; however, many articles suggest that the MB2 canal is particularly common in humans around 25–35 years of age [17, 37]. As the present study is a pilot, our first analyses were performed in patients at this age and two groups were formed of patients aged 21–30 years and 31–40 years.

Our present findings reveal no significant differences in the distribution of the MB2 canal of the maxillary first molars between the two age groups; age was found to have an effect on the incidence of the MB2 canal of the mesiobuccal root only in maxillary second molars. The 31–40 age group showed a greater number of MB2 canals in maxillary second molars than the 21–30 age group. Similarly, in a study of a Chilean population, Abarca et al. [6] observed a higher occurrence of the MB2 canal in the maxillary first and second molars in older patients. In contrast, a study by Zheng et al. [17] showed a significantly greater number of additional MB2 canals among patients between 20 and 30 years of age than among older patients (group aged 30–40 years, 40–50 years, 50–60 years, >60 years) or younger people (group aged 10–20 years). This is in concordance with the results of a study by Neaverth et al. [37].

These differences in study results may be due to the small size of the our sample, and the range of other anatomical forms observed among the second molar teeth apart from only three or four canals.

## Conclusions

Within the limitations of this study, it can be concluded that differences exist in the number and configuration of roots and root canals between maxillary second and first molars in the studied patients. In the second molar teeth, in addition to three-root or four-root canal forms, a few cases of teeth with only one root canal or C-shaped root canals were been found. Due to the anatomical complexity of the mesiobuccal root and the frequent occurrence of the MB2 canal, the endodontist should consider the presence of two canals in this root during treatment. More attention should be given to the detection of additional canals during root canal treatment in maxillary permanent molars, especially during the treatment of the upper first molars or root canal treatment of male patients. These anatomical differences should be taken into account while treating root canals of maxillary molars, as it could influence endodontic treatment.

## Abbreviations

CBCT: Cone Beam Computed Tomography
MB2: Second (additional) mesiobuccal canal

## References

[1] Vertucci FJ (2005). Root canal morphology and its relationship to endodontic procedures. Endod Topics.

[2] Weine FS, Healey HJ, Gerstein H, Evanson L (1969). Canal configuration in the mesiobuccal root of the maxillary first molar and its endodontic significance. Oral Surg Oral Med Oral Pathol..

[3] Ahmad IA, Al-Jadaa A (2014). Three root canals in the mesiobuccal root of maxillary molars: case reports and literature review. J Endod..

[4] Betancourt P, Navarro P, Cantín M, Fuentes R (2015). Cone-beam computed tomography study of prevalence and location of MB2 canal in the mesiobuccal root of the maxillary second molar. Int J Clin Exp Med.

[5] Betancourt P, Navarro P, Muñoz G, Fuentes R. Prevalence and location of the secondary mesiobuccal canal in 1,100 maxillary molars using cone beam computed tomography. BMC Med Imaging. 2016; 10.1186/s12880-016-0168-2. 10.1186/s12880-016-0168-2 PMC5133760 27908285

[6] Abarca J, Gómez B, Zaror C, Monardes H, Bustos L, Cantin M (2015). Assessment of mesial root morphology and frequency of MB2 canals in maxillary molars using cone beam computed tomography. Int J Morphol.

[7] Alrahabi M, Zafar MS (2015). Evaluation of root canal morphology of maxillary molars using cone beam computed tomography. Pak J Med Sci.

[8] Reis AG, Grazziotin-Soares R, Barletta FB, Fontanella VR, Mahl CR (2013). Second canal in mesiobuccal root of maxillary molars is correlated with root third and patient age: a cone-beam computed tomographic study. J Endod..

[9] Blattner TC, George N, Lee CC, Kumar V, Yelton CD (2010). Efficacy of cone-beam computed tomography as a modality to accurately identify the presence of second mesiobuccal canals in maxillary first and second molars: a pilot study. J Endod..

[10] Omer OE, Al Shalabi RM, Jennings M, Glennon J, Claffey NM (2004). A comparison between clearing and radiographictechniques in the study of the root-canal anatomy of maxillary first and second molars. Int Endod J.

[11] Patel S (2009). New dimensions in endodontic imaging: part 2. Cone beam computed tomography. Int Endod J.

[12] Venskutonis T, Plotino G, Juodzbalys G, Mickevičienė L (2014). The importance of cone-beam computed tomography in the management of endodontic problems: a review of the literature. J Endod..

[13] Praveen BN, Shubhasini AR, Bhanushree R, Sumsum PS, Sushma CN (2013). Radiation in dental practice: awareness, protection and recommendations. J Contemp Dent Pract.

[14] Alavi AM, Opasanon A, Ng YL, Gulabivala K (2002). Root and canal morphology of Thai maxillary molars. Int Endod J.

[15] Ng YL, Aung TH, Alavi A, Gulabivala K (2001). Root and canal morphology of Burmese maxillary molars. Int Endod J.

[16] Pattanshetti N, Gaidhane M, Al Kandari AM (2008). Root and canal morphology of the mesiobuccal and distal roots of permanent first molars in a Kuwait population-a clinical study. Int Endod J.

[17] Zheng QH, Wang Y, Zhou XD, Wang Q, Zheng GN, Huang DM (2010). A cone-beam computed tomography study of maxillary first permanent molar root and canal morphology in a Chinese population. J Endod..

[18] Neelakantan P, Subbarao C, Ahuja R, Subbarao CV, Gutmann JL (2010). Cone-beam computed tomography study of root and canal morphology of maxillary first and second molars in an Indian population. J Endod..

[19] Kim Y, Lee S-J, Woo J (2012). Morphology of maxillary first and second molars analyzed by cone-beam computed tomography in a Korean population: variations in the number of roots and canals and the incidence of fusion. J Endod..

[20] Tanvi M, Vimala N, Lalitagauri M (2016). Evaluation of the root morphology of maxillary permanent first and second molars in an Indian subpopulation using cone beam computed tomography. J Dent Med Sci.

[21] Christie WH, Peikoff MD, Fogel HM (1991). Maxillary molars with two palatal roots: a retrospective clinical study. J Endod..

[22] Zhang R, Yang H, Yu X, Wang H, Hul T, PMH D (2011). Use of CBCT to identify the morphology of maxillary permanent molar teeth in a Chinese subpopulation. Int Endod J.

[23] Yang ZP, Yang SF, Lee G (1988). The root and root canal anatomy of maxillary molars in a Chinese population. Endod Dent Traumatol.

[24] Jo HH, Min JB, Hwang HK (2016). Analysis of C-shaped root canal configuration in maxillary molars in a Korean population using cone-beam computed tomography. Restor Dent Endod.

[25] Nikoloudaki GE, Kontogiannis TG, Kerezoudis NP (2015). Evaluation of the root and canal morphology of maxillary permanent molars and the incidence of the second Mesiobuccal root canal in Greek population using cone-beam computed tomography. Open Dent J.

[26] Degerness RA, Bowles WR (2010). Dimension, anatomy and morphology of the mesiobuccal root canal system in maxillary molars. J Endod..

[27] Sert S, Bayirli GS (2004). Evaluation of the root canal configurations of the Mandibular and maxillary permanent teeth by gender in the Turkish population. J Endod..

[28] Gilles J, Reader A (1990). An SEM investigation of the mesiolingual canal in human maxillary first and second molars. Oral Surg Oral Med Oral Pathol.

[29] Al Shalabi RM, Omer OE, Glennon J, Jennings M, Claffey NM (2000). Root canal anatomy of maxillary first and second permanent molars. Int Endod J.

[30] Kulild JC, Peters DD (1990). Incidence and configuration of canal systems in the mesiobuccal root of maxillary first and second molars. J Endod..

[31] Al-Fouzan KS, Ounis HF, Merdad K, Al-Hezaimi K (2013). Incidence of canal systems in the mesio-buccal roots of maxillary first and second molars in Saudi Arabian population. Aust Endod J.

[32] Lee JH, Kim KD, Lee JK, Park W, Jeong JS, Lee Y, Gu Y (2011). Mesiobuccal root canal anatomy of Korean maxillary first and second molars by cone beam computed tomography. Oral Surg Oral Med Oral Pathol Oral Radiol Endod.

[33] Cleghorn BM, Christie WH, Dong CS (2006). Root and root canal morphology of the HumanPermanent maxillary first molar: a literature review. J Endod.

[34] Pecora JD, Woelfel JB, Sousa Neto MD, Issa EP (1992). Morphologic study of the maxillary molars. Part II: internal anatomy. Braz Dent J.

[35] Rouhani A, Bagherpour A, Akbari M, Azizi M, Nejat A, Naghavia N (2014). Cone-beam computed tomography evaluation of maxillary first and second molars in Iranian population: a morphological study. Iran Endod J.

[36] Shetty H, Sontakke S, Karjodkar F, Gupta P, Mandwe A, Banga KS (2017). A cone beam computed tomography (CBCT) evaluation of mb2 canals in endodontically treated permanent maxillary molars. A retrospective study in Indian population. J Clin Exp Dent.

[37] Neaverth EJ, Kotler LM, Kaltenbach RF (1987). Clinical investigation (in vivo) of endodontically treated maxillary first. J Endod.

